# The impact of containment policy and mobility on COVID-19 cases through structural equation model in Chile, Singapore, South Korea and Israel

**DOI:** 10.7717/peerj.15769

**Published:** 2023-08-01

**Authors:** Jun Jiao, Leiyu Shi, Manfei Yang, Junyan Yang, Meiheng Liu, Gang Sun

**Affiliations:** 1 Department of Health Management, School of Health Management, Southern Medical University, Guangzhou, Guangdong, China; 2 School of Sociology and Population Studies, Renmin University of China, Beijing, China; 3 Department of Health Policy and Management, Bloomberg School of Public Health, Johns Hopkins University, Baltimore, MD, United States of America

**Keywords:** COVID-19, Structural equation modeling, Mobility, Containment policies

## Abstract

**Objectives:**

The study aims to understand the impact of containment policy and mobility on COVID-19 cases in Chile, Singapore, South Korea and Israel. To provide experience in epidemic prevention and control.

**Methods:**

Structural equation modeling (SEM) of containment policies, mobility, and COVID-19 cases were used to test and analyze the proposed hypotheses.

**Results:**

Chile, Israel and Singapore adopted containment strategies, focusing on closure measures. South Korea adopted a mitigation strategy with fewer closure measures, focusing on vaccination and severe case management. There was a significant negative relationship among containment policies, mobility, and COVID-19 cases.

**Conclusion:**

To control the COVID-19 and slow down the increase of COVID-19 cases, countries can increase the stringency of containment policies when COVID-19 epidemic is more severe. Thus, countries can take measures from the following three aspects: strengthen the risk monitoring, and keep abreast of the COVID-19 risk; adjust closure measures in time and reduce mobility; and strengthen public education on COVID-19 prevention to motivate citizen to consciously adhere to preventive measures.

## Introduction

COVID-19 still negatively affects normal life. The World Health Organization (WHO) recommends a containment strategy in the first stage of a pandemic, especially by actively tracing and isolating close contacts to prevent their transformation into a chain of transmission, to stop as much transmission as possible, and eventually make the pandemic disappear. As of May 1, 2022, there were 51,352,166 confirmed cases and 6,261,385 deaths worldwide (Centers for Disease Control and Prevention (CDC), 2020).

Chile, Israel, Singapore and South Korea are taking different measures to deal with COVID-19. Chile is a model country in the fight against COVID-19, making full use of big data to aid government decision-making, and of the highest vaccination rates in the world Singapore adopted strict border containment measures in the early stage and initiated a graded diagnosis and treatment policy (Wang et al., 2021b). Singapore used lockdown measures to isolate migrant workers on April 7, 2020 (Chen et al., 2021b). South Korea, in the early stages of COVID-19, implemented a containment strategy, escalated public alert levels, and entry control procedures. In response to cluster outbreaks, South Korea implemented a strict lockdown and 14-day quarantine (Chen et al., 2021a). On April 30, 2022, 330,000 per million people in South Korea had been infected (Mathieu et al., 2020). Israel is a superior student of vaccination, being the first country to fully vaccinate, the first to administer vaccine booster shots and the first to prescribe the fourth dose of vaccine (Andrews et al., 2022; Regev-Yochay et al., 2022). During the Omicron period, Israel was the first country who declared lockdown (Times of Israel, 2023a). But on March 1, 2022, it lifted most of the restrictions on COVID-19, including the “gathering restriction” (Government of Israel, 2023). All four of these countries have adopted containment policies to some extent in response to COVID-19. At present, there are more descriptive articles on national policies, and more policy summaries on countries with better epidemic control, such as Vietnam and China, and countries with the highest number of confirmed cases, such as the United States, India and Brazil (Tran et al., 2020; Le et al., 2021; Ha et al., 2021). In contrast, quantitative studies on containment policies are limited, with only work modeling the effect of social distancing on containment of COVID-19 transmission.

The structural equation model (SEM) is a method to establish, estimate and test the causality model, which can analyze the effect of individual indicators on the whole and the interrelationship between individual indicators (Efron, 2000). In this study, the SEM of containment policies, mobility, and COVID-19 cases was constructed to test and analyze the proposed hypotheses. Specifically, we try to answer one important research question: whether changes in containment policies could impact COVID-19 cases.

## Research Framework

Containment policies for COVID-19 were first implemented in Wuhan, China on January 23, 2020. These policies are collectively known as the Wuhan City containment policy and include home quarantine, strict exit screening measures closure, a shutdown of public transportation, a ban on leaving Wuhan, and other containment policies (Caristia et al., 2020; Kumar, Priya & Srivastava, 2021). Scholars have used a variety of statistical methods and models to analyze the effects of certain periods and geographic closure measures. In studies of early stages of COVID-19, it was shown that conducting a social restriction policy and limiting social distance was effective in reducing COVID-19 cases (Chu et al., 2020; McGrail et al., 2020). Alfano & Ercolano (2020) estimated the impact of lockdown *via* feasible generalized least squares fixed effect. Their results show that lockdown is effective in reducing the number of new cases, and it can keep 20 days. Lau et al. (2020) focused on the lockdown period in Wuhan, during which the increase rate of cases decreased. These studies suggest that social distancing and lockdowns are effective in reducing COVID-19 cases. However, containment policies also received some different results. Quilty et al. (2020) found that airport screening is unlikely to detect a sufficient proportion of 2019-nCoV infected travelers. In addition, some scholars believe that containment policies are not sustainable policies, and long-term use would seriously affect the economy and normal production and life order (Kumar, Priya & Srivastava, 2021). Studies have found that airport screening has had a modest impact on reducing COVID-19 cases, and some containment policies may not be sustainable. What are the effects of containment policies in the face of the long-term epidemic and constant variation of COVID-19? Thus, it was hypothesized that:

### H1 containment policies are negatively correlated with COVID-19 cases

Mobility is the public’s response to the containment policies and the severity of the containment policies. Data of Google Mobility can effectively reflect the implementation of containment policies (Balcan et al., 2009; Charu et al., 2017; Fang, Wang & Yang, 2020; Guo, Deng & Gu, 2021). Many countries use movement restrictions as part of epidemic response (Charu et al., 2017). Morens, Folkers & Fauci (2022) studied the relationship among vaccinations, mobility, and COVID-19 transmission. The study highlights the significance of mobility in realizing the effectiveness of COVID-19 vaccines (Guo, Deng & Gu, 2021). Fan et al. (2020) believed that migration may be the primary reason for the long-distance transmission of the disease, and proposed containment policies. The level of containment measures varies from country and period, with mandatory containment measures restricting access to people. Also, population movements may be exacerbated when containment policies are not in place or when there is a panic effect on the population about the virus (Huang et al., 2020; Fang, Wang & Yang, 2020; Jiao et al., 2022). In this article, we study whether containment policies are related to mobility and how containment policies affect mobility. Thus, the following was hypothesized:

### H2 containment policies are negatively correlated with mobility

In infectious diseases, mobility is strongly correlated with epidemic transmission (Viboud et al., 2006; Balcan et al., 2009). When people move, they take contagious diseases with them and spread them. Since COVID-19 is spread through respiratory droplets and indirect contact, the reduction of mobility and contact reduction can reduce the effective reproduction rate (*R*_t_) and control the disease epidemic. The achievement of *R*_t_ < 1 is necessary to stop the spread of infectious diseases (Yang et al., 2020; Noland, 2021). In most studies, mobility is mostly used as a moderating variable (Quilty et al., 2020; Alfano & Ercolano, 2020; Guo, Deng & Gu, 2021). Through modeling, Jia et al. (2020) found that mobility had a greater impact on COVID-19 spread in Hubei province, but a weaker impact outside Hubei Province. In addition, mobility is considered to be an important factor leading to long-distance infection and prone to cluster outbreaks, which is also an important factor to be paid attention to in the long-term response to COVID-19 (Chen et al., 2020; Chinazzi et al., 2020). Thus, the following was hypothesized.

### H3 mobility is positively correlated with COVID-19 cases

**Figure 1:**
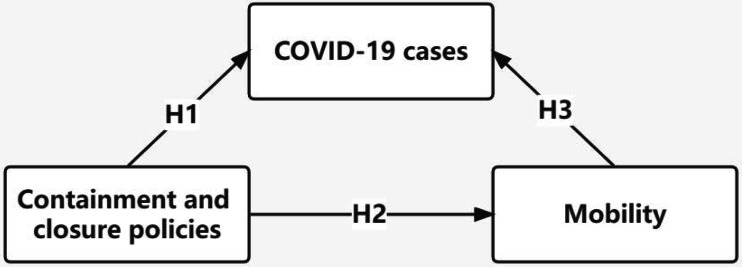
Research framework.

Figure 1 represents the research framework of this study. The novelty of this study is not only the systematic analysis of COVID-19 containment policies, but also conducted an empirical study on the relationship among containment policies, mobility, and COVID-19 cases by constructing SEM.

## Methods

### Variables and sources

There are three parts of data in the study including containment policies, mobility, and COVID-19 cases in Chile, Singapore, South Korea and Israel.

Data on containment policies from Oxford COVID-19 Government Response Tracker (https://github.com/OxCGRT/covid-policy-tracker). All containment policies were used including eight categories: school closing, workplace closing, cancel public events, restrictions on gathering size, closed public transport, stay-at-home requirement, restrictions on internal movement, and international travel controls and assigns a value of 0-5 according to their severity.

These Community Mobility Reports aimed to provide insights into what changed in response to policies aimed at combating COVID-19. The mobility data at the country level wais collected from Community Mobility Reports provided by Google (https://ourworldindata.org/covid-google-mobility-trends), which uses anonymized data provided by apps such as Google Maps, the company has produced a regularly updated dataset that shows how peoples’ movements have changed throughout the pandemic. And reports population mobility data for six locations: retail and recreation, grocery and pharmacy, residential, transit stations, parks, and workplaces. In this work, we consider all place categories provided, and mobility has changed compared to baseline days (the median value for the 5 weeks from January 3 to February 6, 2020), and we collected 730 population mobility data for every four countries.

Data on COVID-19 cases are from Our World in Data (https://ourworldindata.org/coronavirus).

Thus, we collected data on containment policies, mobility, and COVID-19 cases in Chile, Singapore, South Korea and Israel from May 1, 2020, to April 30, 2022. Then, we took a set of data every six days. In the end, a total of 488 sets of data were obtained.

### Structural equation modeling

SEM was used to measure the causal relationships between latent variable constructs. It was run by using AMOS 24. This study considered using SEM in constructing a model of the impact of containment policies-mobility-COVID-19 cases.

Firstly, according to the relevant theories and literature, the theoretical hypothesis and model of containment policies—mobility—COVID-19 cases in the COVID-19 were initially constructed. Secondly, we analyzed the data and carried out empirical research to analyze and verify the theoretical model and research hypothesis. Finally, the containment policies—mobility—COVID-19 cases are concluded.

## Results

### National trend of COVID-19 pandemic

In this part, this study describes the trends of containment policies, mobility and COVID-19 cases, which can quickly discover the point and degree of change based on time and clearly understand the differences in the four countries.

**Figure 2:**
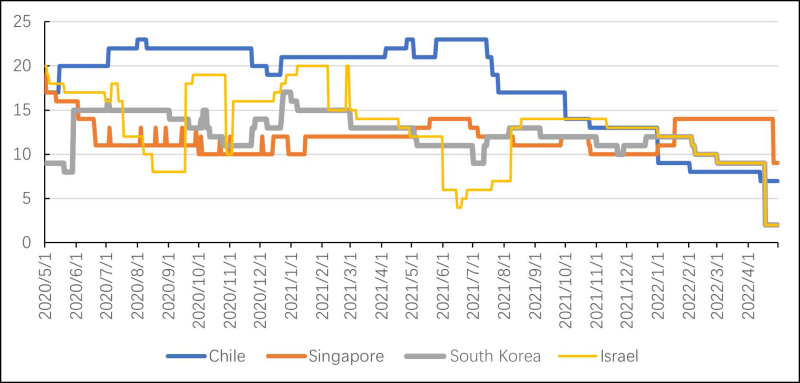
Trend of containment policies.

As shown in Fig. 2, trends of containment policies in four countries show an overall decline and phase fluctuations, which is generally consistent with the timing of SARS-CoV-2 variants in countries. The total data in Chile is higher than in three other countries, especially in the early period of COVID-19. Chile closed public places and workplaces for a long time, canceling events, limiting transportation, keep closing schools until sanitary conditions allow a gradual return. Singapore’s trend is relatively smooth. The changes in containment policies are small and return to the original state after a phased increase. Workplace closing and international travel controls overall remained generally unchanged. In the beginning, South Korea’s containment policies were at a low level, and only school closing and international travel control measures were taken. And public transport has never been shut down. South Korea only retained international travel controls after April 18, 2022. Israel’s trend fluctuates greatly, showing a trend of rapid rise and fall. The policy changes come from three policy changes: cancel public events, stay at home requirements, and internal mobility restrictions.

**Figure 3:**
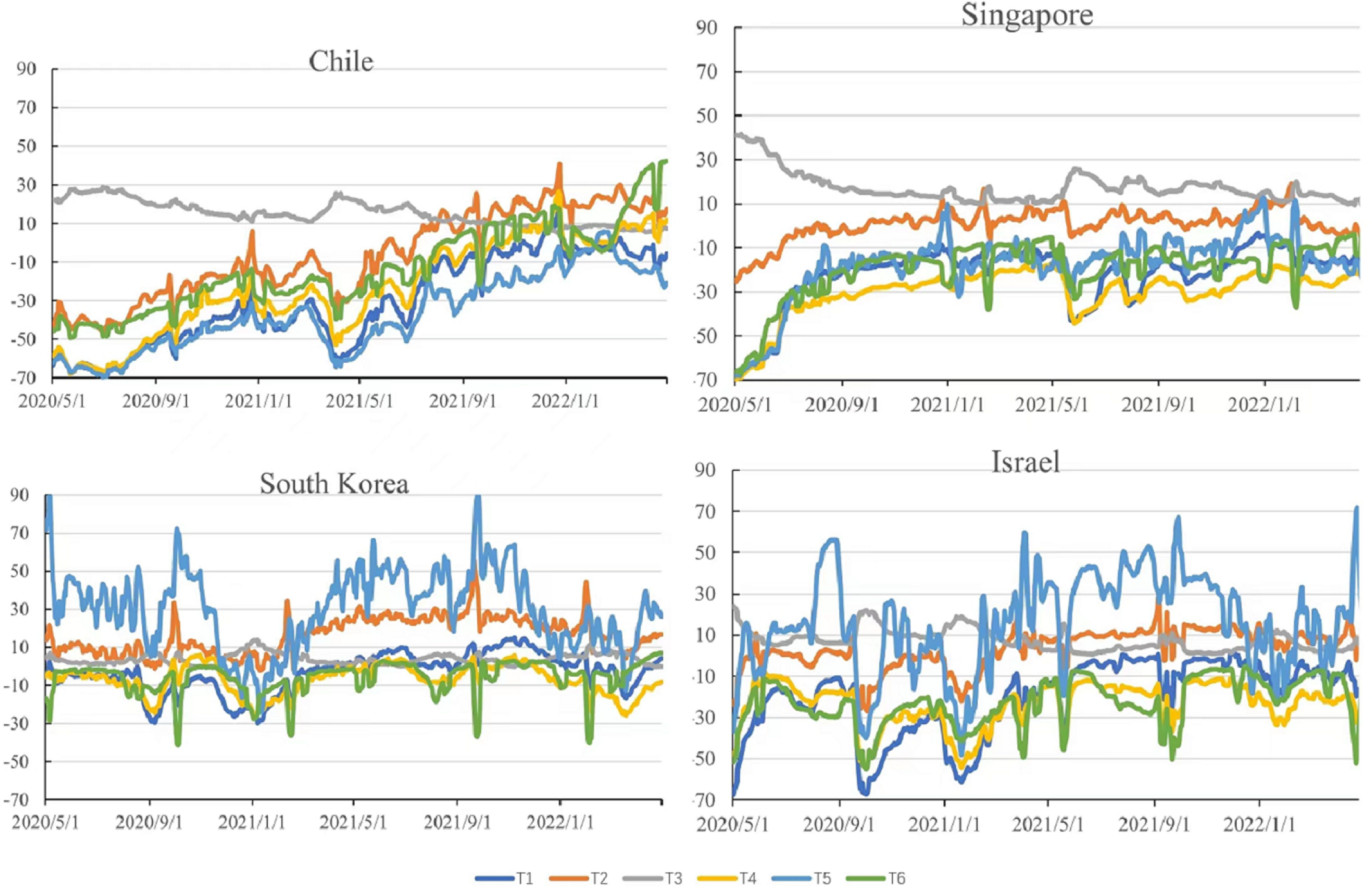
Trend of mobility in Chile, Singapore, South Korea, and Israel (compared to baseline days—the median value for the 5 weeks from January 3 to February 6, 2020).

As shown in Fig. 3, T1 to T6 are mobility data of places in retail and recreation, grocery and pharmacy, residential, transit stations, parks, and workplaces. For T1-retail and recreation, most of the time, all four countries were below the baseline, showing an overall upward trend. However, Chile in December 2021 and South Korea in June and December 2021 are above the baseline. For T2-grocery and pharmacy, there are many differences among four countries. For T3-residential, Chile, Singapore and the early period of Israel were higher than baseline, and in others has no obvious change compared with baseline. For T4-transit stations, others are below the baseline, except for Chile which is above the baseline after October 2021. For T5-parks, there are two trends. In Chile and Singapore, the trend of T5 is lower than baseline. However, in South Korea and Israel, trends are wild fluctuation and great higher than baseline, especially in South Korea. And the trend of T6-workplaces is similar to T4.

**Figure 4:**
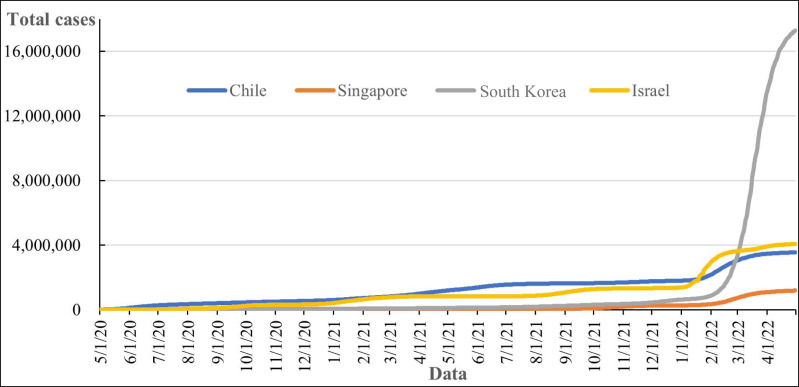
Total cases of COVID-19.

As shown in Fig. 4, the rank of total cases in South Korea, Israel, Chile, and Singapore. For total cases per million people, Chile is 185,226.106 cases per million people, Singapore is 218,800.43 cases per million people, South Korea is 336,723.26 cases per million people, Israel is 438,715.32 cases per million people.

### Structural equation modeling

SEM is a method of latent variable analysis that offers a high degree of flexibility in terms of modeling methods for applied research questions (Hall & Clark, 2023). SEM was used to research to understand the complex relationship of containment policies, mobility and COVID-19 related factors and to assist in formulating effective containment policies to promote policy and practice.

In this study, the Pearson correlation analysis was used to examine the relationship between latent variables used within the research scope, and the results were presented in Table 1. A significant correlation was observed among the three (*P* < 0.001). There was a negative correlation between containment policies and mobility (*r* =  − 0.662, *P* = 0.00 < 0.001) and cumulative COVID-19 cases (*r* =  − 0.276, *P* = 0.00 < 0.001). There was a positive correlation between mobility and COVID-19 cases (*r* = 0.176, *P* = 0.00 < 0.001). The two variables that were found to have a higher relationship level than other variables in the study were containment policies related to mobility. The relationship between these two variables was seen to be a positive correlation and moderate correlation (*r* =  − 0.662, *P* = 0.00 < 0.001). Furthermore, the weakest positive relationship between the variables was found between mobility and cumulative COVID-19 cases (*r* = 0.176, *P* = 0.00 < 0.001).

**Table 1:** Correlation values between scales.

Scale	Containment policies	Mobility	COVID-19 cases
Containment policies	1	−0.66^***^	−0.28^***^
Mobility		1	0.18^***^
COVID-19 cases			1

**Notes.**

*** Indicates that a correlation is significant at a level of *p* < 0.001.

**Figure 5:**
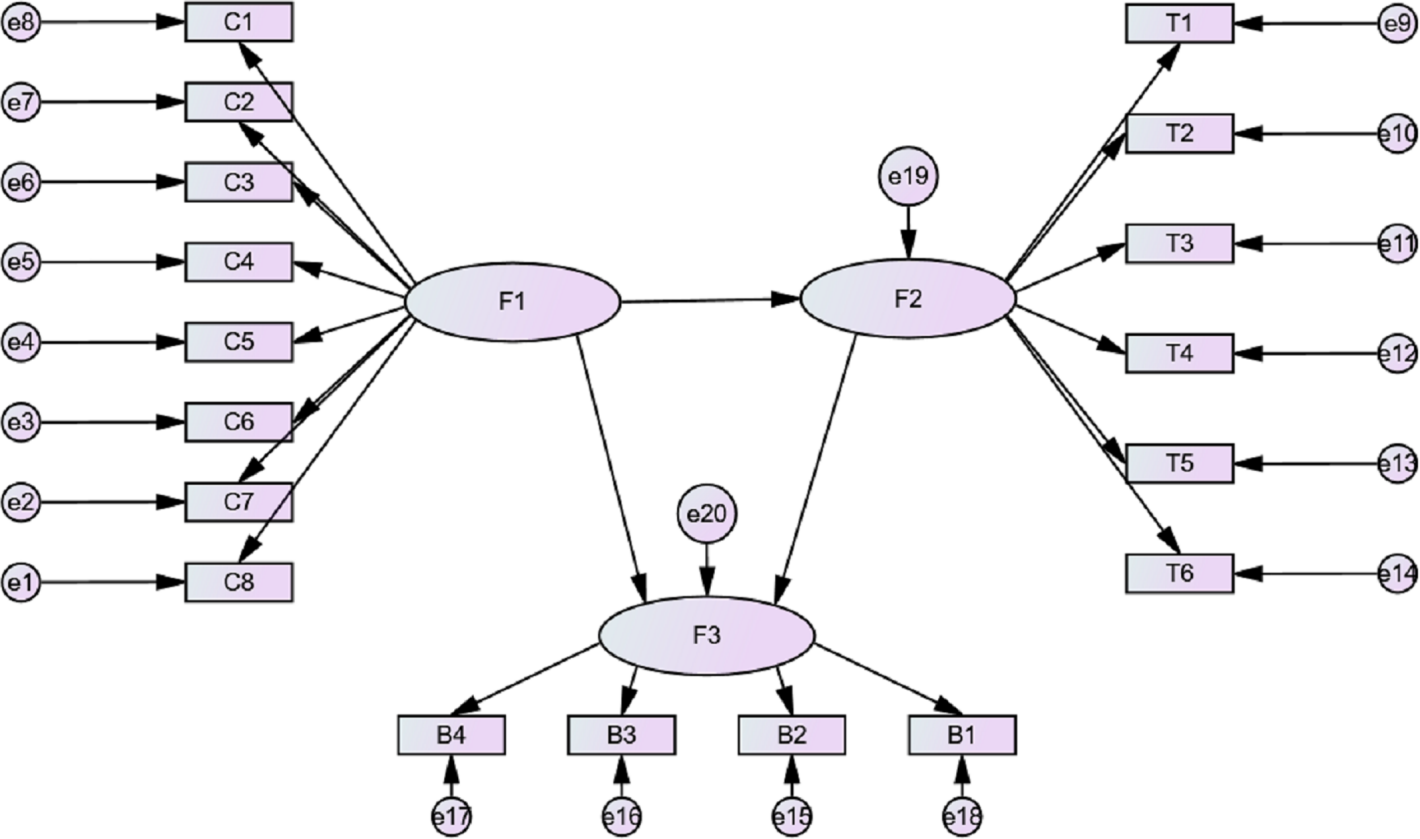
Initial SEM model in this study.

Then the initial SEM is tested. As shown in Fig. 5, the results (*χ*2/SD = 24.76, GFI = 064, AGFI = 0.53, NFI = 0.64, RMSEA = 0.22, CFI = 0.65) did not meet the standard and the model should be corrected.

In correcting the SEM, we tested the measurement models. After model modification, mobility and COVID-19 cases retained three factors respectively, which was exact identification, while containment policies retained four factors, which showed excellent fitting levels (*χ*^2^/SD = 1.78, GFI = 0.99, AGFI = 0.98, NFI = 0.99, RMSEA = 0.04, CFI = 0.99) (Iacobucci, 2010).

In this stage, the impact of containment policies, mobility, and cumulative COVID-19 cases was investigated through SEM. Whether or not the measurement models were validated was then analyzed with Chi-square , *χ*^2^/SD, GFI, AGFI, NFI, RMSEA, and CFI fit indices.

**Figure 6:**
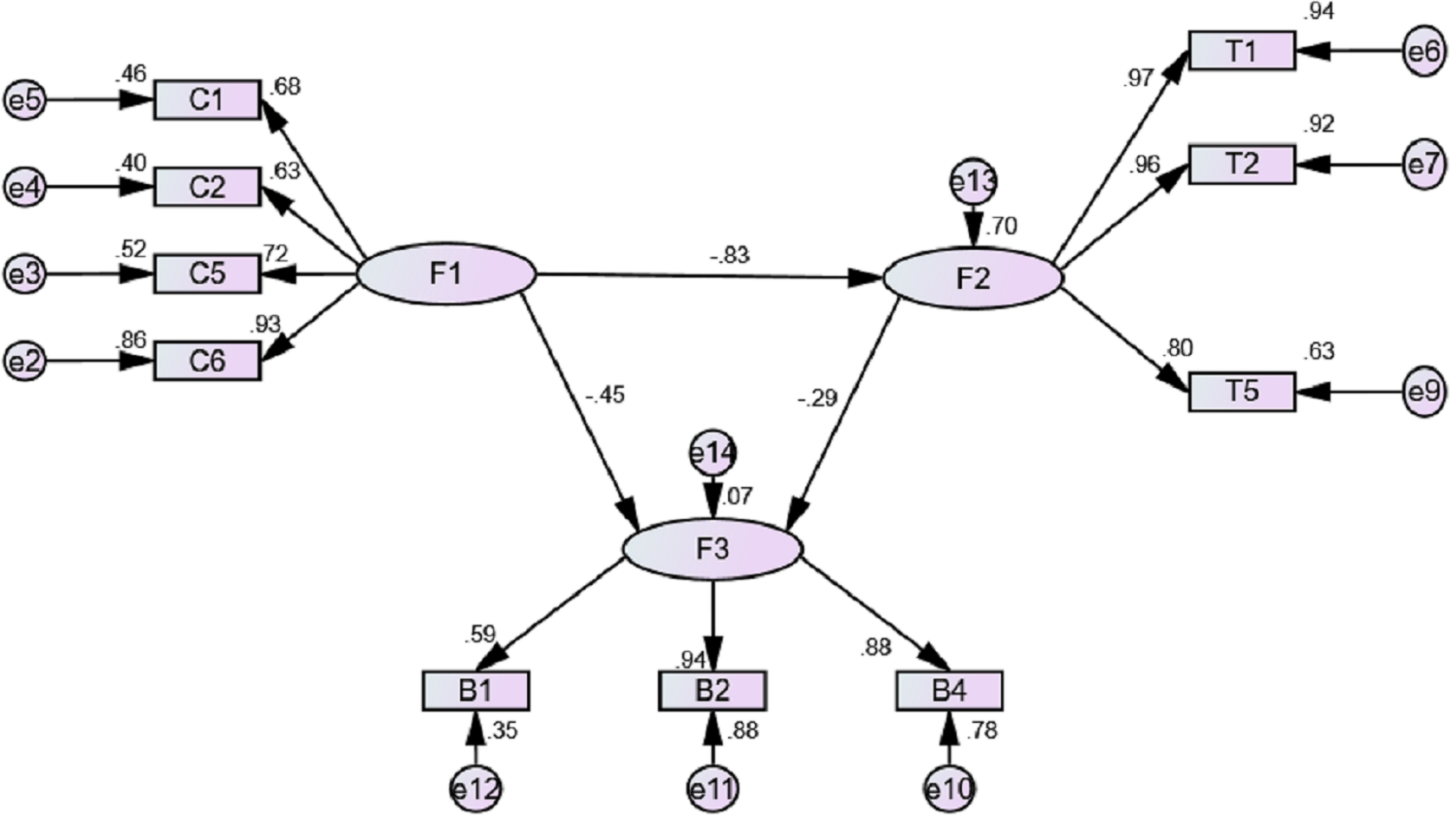
Final SEM model in this study.

In the testing of the final model, the paths between the variables and the model were found to be significant at the 0.001 level. The fit indices of the hypothetical model were calculated as *χ*2/SD = 9.26. Moreover, it was determined to have acceptable values of GFI = 0.91, AGFI = 0.84, NFI = 0.92, RMSEA = 0.13, and CFI = 0.93. These values reveal that the fit indices of the model can be considered both good and within the acceptable range. The final SEM model is presented in Fig. 6.

Moreover, Table 2 presents the variance values, standard error, P, and standardized regression coefficients explained in the model regarding direct affection. After correction, all paths pass the significance test.

**Table 2:** Revised model path analysis results.

Path		Unstd.	Std.	S.E.	*P*
F2	F1	−25.49	−0.84	1.60	^***^
F3	F2	−17696.80	−0.29	6248.70	0.005
F3	F1	−831755.49	−0.45	203669.52	^***^
F1	C1	1	0.68	0.09	^***^
	C2	0.76	0.63	0.05	^***^
	C5	0.71	0.72	0.06	^***^
	C6	1.56	0.93		
F2	T1	1	0.97		
	T2	0.81	0.96	0.02	^***^
	T5	1.40	0.80	0.05	^***^
F3	B1	1	0.60		
	B2	0.04	0.94	0.01	^***^
	B4	0.01	0.88	0.00	^***^

**Notes.**

*** A correlation is significant at a level of *p* < 0.001.

The standardized regression coefficient between containment policies and mobility was found to be −0.84. This value indicates that there is a negative correlation between them. Moreover, containment policies are a strong influence factor on mobility. This result confirms the first hypothesis of the study (H1: Containment policies are negatively correlated with COVID-19 cases). The standardized regression coefficient between mobility and COVID-19 cases was found to be −0.29, which is a weak negative correlation. In other words, the regression weight for mobility in the prediction of COVID-19 cases is at the 0.01 level. This result is inconsistent with the third hypothesis of the study (H3: Mobility is positively correlated with COVID-19 cases). The standardized regression coefficient between containment policies and COVID-19 cases was found to be −0.45. This result indicated that there was a moderate negative correlation between containment policies and COVID-19 cases found. This result confirms the second hypothesis of the study (H2: Containment policies are negatively correlated with mobility).

## Discussion

This study investigated the containment policies, mobility, and COVID-19 cases in Chile, Singapore, South Korea and Israel in response to COVID-19, to provide experience and lessons learned for studying the impact of containment policies on mobility and COVID-19, and for better response to COVID-19. Chile, Israel and Singapore adopted containment strategies, which aim to prevent spread of infections and prefer social intervention measures to block epidemics spread (Jiao et al., 2022). Chile strictly managed population management and border closures. Israel focused on entry control, and Singapore adopted a regular blockade. In contrast, South Korea adopted a mitigation strategy, which aims to reduce the pandemic impact, and it consistently refrained from a large-scale closure policy and focused on vaccines and severe cases.

In addition, SEM of containment policies, mobility, and COVID-19 cases was used to test and analyze the proposed hypotheses. In COVID-19, containment policies, mobility, and COVID-19 cases were significantly and negatively related to each other. This article discusses the three previously proposed hypotheses and provides targeted recommendations for responding to COVID-19.

### National Containment policies, mobility, and COVID-19 cases

In response to COVID-19, four countries have adopted different containment policies. As the Community Mobility Reports write on their home page: these Community Mobility Reports aimed to provide insights into what mobility changed in response to policies aimed at combating COVID-19. COVID-19 spreads between humans through respiratory droplets produced when an infected person coughs or sneezes (Pathania et al., 2021). Population concentrations speed up COVID-19 spreading and are leading to more cases. Mobility reflects the extent to which people are implementing policies and is reflected in COVID-19 case data. In this study, Chile, Israel and Singapore adopted containment strategies, focusing on the effect of lockdown measures and social restriction policies, which used lockdown to block epidemic spread. South Korea has adopted a mitigation strategy that focuses less on lockdown and more on vaccination and severe case management.

In the early stages of COVID-19, Chile attached importance to containment policies and focused on population management and border closing. At the height of the COVID-19 epidemic, home quarantine was mandatory when Chile announced into “national disaster—a state of the nation in difficult times”. In the global push for vaccination, Chile has seized the opportunity to accelerate vaccination far faster than any other country in the Americas (Castillo, Villalobos Dintrans & Maddaleno, 2021; World Health Organization (WHO), 2022). However, since Chile began mass vaccination in February 2021, mobility and COVID-19 cases have increased. For these reasons, some academics suggested that the rapid and effective vaccination campaign gave the Chilean government and people a false sense of security, lifting closure measures too early and causing too many people to traveling (National Public Radio (NPR), 2021; Taylor, 2021).

Like Chile, Israel is a vaccination star (Andrews et al., 2022). Israel is very sensitive to the use of containment policies, that is, policies are very variable and change with COVID-19. Israel has also seen the biggest change in blockade policy among the four countries. Among the containment policies, Israel has focused on entry control measures. Since Israel is a small country, most arrivals go through its only international airport, making entry control measures easier to implement (Goldberg et al., 2021). Due to the rapid change of containment policies, Israel used the Traffic Light Model to show the epidemic level in the region, so that the public could learn about it in time. The index of the Traffic Light Model is updated once every two weeks. The index is calculated by weighing the number of new cases, the percentage of positive test results, and infection rate. There are four scales, green, yellow, orange and red, with the strict rising in that order (Local Councils Traffic Light Model, 2023).

However, the Israel epidemic began to increase rapidly in 2022. On March 1, 2022, Israel announced the full opening of its borders, leading to an increase in COVID-19 cases. “After more than two years of COVID-19, it is time to return to a more normal reality—living with this virus and taking the appropriate steps to protect public health”. Israeli Prime Minister Bennett said in a statement that according to “all the data, the epidemic is steadily declining (Israel National News, 2023b)”.

Singapore responded quickly in early stages of COVID-19, and focused on containment policies, preventing imported cases through a strict border policy (Tan et al., 2021). All these restricting policies curb mobility. After the outbreak phase, Singapore’s containment policies were basically unchanged. The trend of containment policies is much lower than those of the other three countries. Their mobility is largely below the baseline, except for residential. Why have singapore’s containment policies not changed much compared to the other countries? Because even though Singapore does not implement strict containment policies, mobility did not surge rapidly but steadily and slowly increased. This is closely related to the awareness of health education and a clear understanding of COVID-19 in Singapore. For Singapore, it is appropriate to the regular lockdown measures (Wang et al., 2021b).

South Korea implemented a mitigation strategy in response to COVID-19. There was not any large-scale lockdown in South Korea. For containment policies, South Korea mainly maintained school closing and international travel controls. In the early stages of COVID-19, South Korea launched a massive campaign to raise public awareness; through extensive and rapid testing; only severe cases admitted and kept a low-level COVID-19 cases. The turning point of COVID-19 in South Korea came in 2022. The Omicron epidemic came as South Korea relaxed quarantine measures, easing border controls and pushing home quarantine policies. Polls in South Korea show that majority support easing them, and there were gatherings for people infected with COVID-19. South Korea was facing multiple pressures such as a severe shortage of medical resources, public response and an economic downturn. In this case, South Korea has further eased containment policies, and the daily COVID-19 cases became the highest in the world.

### SEM model of containment policies, mobility, and COVID-19 cases

Through the testing and revision of the model, the study found that there was a significant negative relationship among containment policies, mobility, and COVID-19 cases. In addition, the model shows that mobility and COVID-19 cases contradict the hypothesis.

The results of the final SEM model are explained here. In the final SEM model, containment policies retain school closing, workplace closing, close public transport, and stay-at-home requirement: all of the four factors are policies to limit outgoings. In mobility, the three factors of the data of retail and recreation, grocery and pharmacy, and parks were retained, showing different trends in the four countries. COVID-19 cases retain the three factors of total cases, new cases, and new cases per million people, which not only retain the overall case indicators but also pay attention to changing trends and national population data.

### Containment policies and COVID-19 cases

The results of this study point out that, there is a strong negative correlation between containment policies and COVID-19 cases. That is, COVID-19 cases decrease as the severity of containment policies increases. In response to the sudden COVID-19, many countries began to implement travel restrictions and border prevention and control policies, which are also effective ways to reduce COVID-19 cases entering the country (Quilty et al., 2020; Pham et al., 2021). Among containment policies included in the final model, the effect of the stay-at-home requirement (C6) on containment policies was the most significant, with factor loading reaching 0.93 under normalization.

The stay-at-home requirement policy includes curfew, home isolation, banned for unessential activities, *etc*., which is also the most changed policy among containment policies of the four countries. Its audience is all citizens, which is also one of the important policies that require whole citizen, such as India’s time-limited curfews, and China’s silent management (Paital, Das & Parida, 2020; Wang et al., 2021a). The school closing, workplace closing and close public transport policies are all closed policies targeted at specific regions, which only as temporary measures to reduce virus transmission from confined spaces. However, COVID-19 is still in a long-term trend, and prolonged containment policies harm social and economic development (Atim et al., 2021). Even so, we cannot deny the effect of containment policies on controlling COVID-19. Under such circumstances, the country should take appropriate containment policies following the trend of COVID-19, and implement relaxed containment policies that are easy to implement when COVID-19 enters into the normal period, so that COVID-19 prevention and control and economic development should be carried out simultaneously. This requires real-time monitoring and analysis of COVID-19 situation at home and abroad, and timely adjustment of the severity of policies.

### Containment policies and mobility

There is a moderate negative correlation between containment policies and mobility, that is, mobility decreases with the increase in the severity of containment policies. Meanwhile, containment policies are useful in government response to COVID-19 and people are highly compliant. In the early stage of COVID-19, when the pathogenicity, transmission, mortality, and characteristics of virus are not clear, there was a certain fear of COVID-19, and people tended to obey the policy and reduce mobility. And the poor are more vulnerable to COVID-19 (Papageorge et al., 2021; Wang et al., 2021c). In addition, migration may be the primary reason for the long-distance transmission of the disease (Fan et al., 2020). Mobility reflects the influence of the stay-at-home requirement policy through T3-Residential. The residential mobility in Chile and Singapore are both above the baseline, which shows that they implement strict stay-at-home requirement policies.

Strong containment policies can effectively reduce mobility. Research by Yechezkel et al. (2021) also points out that in Israel closure policies have had a marked effect on mobility, especially the elderly. Perceptions of epidemic risk also play a role in their response behavior. The more people perceive a higher epidemic risk, the more they tend to take protective measures. When some countries began to downplay COVID-19 in 2021, reduce the stringency of containment policies, and accelerate the resumption of work and production, the population’s perception of the epidemic risk began to shift. Response behaviors showed by mobility have also changed, as evidenced by increased mobility in public places. Also, a long-term containment policy would generate a backlash (Famiglietti & Leibovici, 2022; Reis et al., 2022). The government should strictly review the publicity of COVID-19 and policies, and enhance public education, which enable the citizen to consciously implement the containment policy and reduce mobility.

### Mobility and COVID-19 cases

There was a weak negative correlation between mobility and COVID-19 cases; that is, the number of COVID-19 cases decreased with increased mobility. This is contrary to our hypothesis H3. Li et al. (2021) suggests that mobility may not always directly affect COVID-19 trends. For this reason, this study identifies a significant change in mobility of some countries after 2021. In South Korea and Israel, park mobility figures remain high and much higher than at-home mobility. Stepping into the Omicron epidemic, the proportion of asymptomatic infections increases, and combined with the immune escape nature of the Omicron variant, it is difficult to detect the virus with a single nucleic acid test (Kannan, Shaik Syed Ali & Sheeza, 2021; Bruel et al., 2022). The population perceives itself to be asymptomatic and delays or even does not seek medical care, which may lack case data but is exacerbated by population mobility. In addition, in the Pearson correlation test, mobility and COVID-19 cases were positively correlated. In the Pearson correlation, the data on mobility included data from public places and also from residential; however, the SEM screened three factors. We believe this may be the reason for the contradictory paths of the Pearson correlation and the SEM.

In this study, SEM was used to quantitatively analyze containment policies, mobility and COVID-19 cases in four countries, Chile, Singapore, South Korea and Israel. The changes during Omicron are clearly explained and analyzed. However, there are still some deficiencies in this study. As COVID-19 is changing all the time, containment policies and population mobility during Omicron were affected before, so the description of this part is insufficient. Another limitation is containment policies in the study present different characteristics in transmission speed and variations in pathogenicity were poorly considered. In future research, we will pay more attention to the policy evolution and influencing factors.

## Conclusion

The study used the SEM to analyze the impact of containment policy and mobility on COVID-19 cases in Chile, Singapore, South Korea and Israel. Chile, Israel and Singapore adopted containment strategies, focusing on the effect of containment policies. South Korea adopted a mitigation strategy that focuses less on containment policies and more on vaccination and severe case management. In COVID-19, there is a significant negative relationship among containment policies, mobility, and COVID-19 cases.

This study was conducted for controlling COVID-19 and slow down the increase of COVID-19 cases, countries can increase the stringency of containment policies when COVID-19 epidemic is more severe. Thus, countries can take measures from the following three aspects: firstly, strengthen the risk monitoring, such as establishing an international epidemic surveillance network; secondly, keep abreast of the COVID-19 risk, adjust closure measures in time and reduce mobility, which can do this by setting up decision trees; thirdly, strengthen public education on COVID-19 prevention to motivate citizen to consciously adhere to preventive measures by social speeches and television advertisements. These policies are fundamental to the management of COVID-19. Management of containment policies, mobility reduction and prediction are needed to address similar future challenges.

##  Supplemental Information

10.7717/peerj.15769/supp-1 Data S1 Data of containment policy and mobility on COVID-19 cases

10.7717/peerj.15769/supp-2 Appendix S1 Measurement model modification

## Additional Information and Declarations

### Data Availability

The following information was supplied regarding data availability: The datasets analyzed during the current study are available at the Oxford COVID-19 Government Response Tracker (https://github.com/OxCGRT/covid-policy-tracker), Google (https://ourworldindata.org/covid-google-mobility-trends), and Our World in Data (https://ourworldindata.org/coronavirus).

## References

[ref-1] Alfano V, Ercolano S (2020). The efficacy of lockdown against COVID-19: a cross-country panel analysis. Applied Health Economics and Health Policy.

[ref-2] Andrews N, Stowe J, Kirsebom F, Toffa S, Rickeard T, Gallagher E, Gower C, Kall M, Groves N, O’Connell A-M, Simons D, Blomquist PB, Zaidi A, Nash S, Iwani Binti Abdul Aziz N, Thelwall S, Dabrera G, Myers R, Amirthalingam G, Gharbia S, Barrett JC, Elson R, Ladhani SN, Ferguson N, Zambon M, Campbell CNJ, Brown K, Hopkins S, Chand M, Ramsay M, Lopez Bernal J (2022). Covid-19 vaccine effectiveness against the Omicron (B.1.1.529) variant. The New England Journal of Medicine.

[ref-3] Atim MG, Kajogoo VD, Amare D, Said B, Geleta M, Muchie Y, Tesfahunei HA, Assefa DG, Manyazewal T (2021). COVID-19 and health sector development plans in Africa: the impact on maternal and child health outcomes in Uganda. Risk Management and Healthcare Policy.

[ref-4] Balcan D, Colizza V, Gonçalves B, Hu H, Ramasco JJ, Vespignani A (2009). Multiscale mobility networks and the spatial spreading of infectious diseases. Proceedings of the National Academy of Sciences of the United States of America.

[ref-5] Bruel T, Hadjadj J, Maes P, Planas D, Seve A, Staropoli I, Guivel-Benhassine F, Porrot F, Bolland W-H, Nguyen Y, Casadevall M, Charre C, Péré H, Veyer D, Prot M, Baidaliuk A, Cuypers L, Planchais C, Mouquet H, Baele G, Mouthon L, Hocqueloux L, Simon-Loriere E, André E, Terrier B, Prazuck T, Schwartz O (2022). Serum neutralization of SARS-CoV-2 Omicron sublineages BA.1 and BA.2 in patients receiving monoclonal antibodies. Nature Medicine.

[ref-6] Caristia S, Ferranti M, Skrami E, Raffetti E, Pierannunzio D, Palladino R, Carle F, Saracci R, Badaloni C, Barone-Adesi F, Belleudi V, Ancona C, AIE working group on the evaluation of the effectiveness of lockdowns (2020). Effect of national and local lockdowns on the control of COVID-19 pandemic: a rapid review. Epidemiologia E Prevenzione.

[ref-7] Castillo C, Villalobos Dintrans P, Maddaleno M (2021). The successful COVID-19 vaccine rollout in Chile: Factors and challenges. Vaccine: X.

[ref-8] Centers for Disease Control and Prevention (CDC) (2020). COVID Data Tracker. https://covid.cdc.gov/covid-data-tracker.

[ref-9] Charu V, Zeger S, Gog J, Bjørnstad ON, Kissler S, Simonsen L, Grenfell BT, Viboud C (2017). Human mobility and the spatial transmission of influenza in the United States. PLOS Computational Biology.

[ref-10] Chen H, Shi L, Zhang Y, Wang X, Sun G (2021a). Policy disparities in response to COVID-19 between China and South Korea. Journal of Epidemiology and Global Health.

[ref-11] Chen H, Shi L, Zhang Y, Wang X, Sun G (2021b). A cross-country core strategy comparison in China, Japan, Singapore and South Korea during the early COVID-19 pandemic. Globalization and Health.

[ref-12] Chen S, Yang J, Yang W, Wang C, Bärnighausen T (2020). COVID-19 control in China during mass population movements at New Year. The Lancet.

[ref-13] Chinazzi M, Davis JT, Ajelli M, Gioannini C, Litvinova M, Merler S, Piontti APastorey, Mu K, Rossi L, Sun K, Viboud C, Xiong X, Yu H, Halloran ME, Longini IM, Vespignani A (2020). The effect of travel restrictions on the spread of the 2019 novel coronavirus (COVID-19) outbreak. Science.

[ref-14] Chu DK, Akl EA, Duda S, Solo K, Yaacoub S, Schünemann HJ (2020). Physical distancing, face masks, and eye protection to prevent person-to-person transmission of SARS-CoV-2 and COVID-19: a systematic review and meta-analysis. Lancet.

[ref-15] Efron B (2000). The bootstrap and modern statistics. Journal of the American Statistical Association.

[ref-16] Famiglietti M, Leibovici F (2022). The impact of health and economic policies on the spread of COVID-19 and economic activity. European Economic Review.

[ref-17] Fan C, Cai T, Gai Z, Wu Y (2020). The relationship between the migrant population’s migration network and the risk of COVID-19 transmission in China—empirical analysis and prediction in prefecture-level cities. International Journal of Environmental Research and Public Health.

[ref-18] Fang H, Wang L, Yang Y (2020). Human mobility restrictions and the spread of the Novel Coronavirus (2019-nCoV) in China. Journal of Public Economics.

[ref-19] Goldberg Y, Mandel M, Bar-On YM, Bodenheimer O, Freedman L, Haas EJ, Milo R, Alroy-Preis S, Ash N, Huppert A (2021). Waning immunity after the BNT162b2 vaccine in Israel. The New England Journal of Medicine.

[ref-20] Government of Israel (2023). Changes to the green pass policy. https://www.gov.il/en/Departments/news/04022022-02.

[ref-21] Government of Israel (2023). Local Councils Traffic Light Model. https://corona.health.gov.il/en/ramzor/.

[ref-22] Guo J, Deng C, Gu F (2021). Vaccinations, mobility and COVID-19 transmission. International Journal of Environmental Research and Public Health.

[ref-23] Ha BTT, Quang LN, Thanh PQ, Duc DM, Mirzoev T, Bui TMA (2021). Community engagement in the prevention and control of COVID-19: insights from Vietnam. PLOS ONE.

[ref-24] Hall GJ, Clark KN (2023). Demystifying longitudinal data analyses using structural equation models in school psychology. Journal of School Psychology.

[ref-25] Huang C, Wang Y, Li X, Ren L, Zhao J, Hu Y, Zhang L, Fan G, Xu J, Gu X, Cheng Z, Yu T, Xia J, Wei Y, Wu W, Xie X, Yin W, Li H, Liu M, Xiao Y, Gao H, Guo L, Xie J, Wang G, Jiang R, Gao Z, Jin Q, Wang J, Cao B (2020). Clinical features of patients infected with 2019 novel coronavirus in Wuhan, China. The Lancet.

[ref-26] Iacobucci D (2010). Structural equations modeling: fit indices, sample size, and advanced topics. Journal of Consumer Psychology.

[ref-27] Israel National News (2023b). Time has come to return to normal reality, to live with the virus—7. https://www.israelnationalnews.com/news/322604.

[ref-28] Jia JS, Lu X, Yuan Y, Xu G, Jia J, Christakis NA (2020). Population flow drives spatio-temporal distribution of COVID-19 in China. Nature.

[ref-29] Jiao J, Shi L, Zhang Y, Chen H, Wang X, Yang M, Yang J, Liu M, Sun G (2022). Core policies disparity response to COVID-19 among BRICS countries. International Journal for Equity in Health.

[ref-30] Kannan S, Shaik Syed Ali P, Sheeza A (2021). Omicron (B.1.1.529)—variant of concern—molecular profile and epidemiology: a mini review. European Review for Medical and Pharmacological Sciences.

[ref-31] Kumar A, Priya B, Srivastava SK (2021). Response to the COVID-19: understanding implications of government lockdown policies. Journal of Policy Modeling.

[ref-32] Lau H, Khosrawipour V, Kocbach P, Mikolajczyk A, Schubert J, Bania J, Khosrawipour T (2020). The positive impact of lockdown in Wuhan on containing the COVID-19 outbreak in China. Journal of Travel Medicine.

[ref-33] Le T-AT, Vodden K, Wu J, Atiwesh G (2021). Policy responses to the COVID-19 pandemic in Vietnam. International Journal of Environmental Research and Public Health.

[ref-34] Li Y, Li M, Rice M, Zhang H, Sha D, Li M, Su Y, Yang C (2021). The impact of policy measures on human mobility, COVID-19 cases, and mortality in the US: a spatiotemporal perspective. International Journal of Environmental Research and Public Health.

[ref-35] Mathieu E, Ritchie H, Rodés-Guirao L, Appel C, Giattino C, Hasell J, Macdonald B, Dattani S, Beltekian D, Ortiz-Ospina E, Roser M (2020). Coronavirus pandemic (COVID-19). Our World in Data. https://ourworldindata.org/coronavirus.

[ref-36] McGrail DJ, Dai J, McAndrews KM, Kalluri R (2020). Enacting national social distancing policies corresponds with dramatic reduction in COVID19 infection rates. PLOS ONE.

[ref-37] Morens DM, Folkers GK, Fauci AS (2022). The concept of classical herd immunity may not apply to COVID-19. Journal of Infectious Diseases.

[ref-38] National Public Radio (NPR) (2021). Chile sees new wave of COVID-19 infections despite rapid vaccine distribution. https://www.npr.org/2021/04/04/984203749/chile-sees-new-wave-of-covid-19-infections-despite-rapid-vaccine-distribution.

[ref-39] Noland RB (2021). Mobility and the effective reproduction rate of COVID-19. Journal of Transport & Health.

[ref-40] Paital B, Das K, Parida SK (2020). Inter nation social lockdown versus medical care against COVID-19, a mild environmental insight with special reference to India. The Science of the Total Environment.

[ref-41] Papageorge NW, Zahn MV, Belot M, Van den Broek-Altenburg E, Choi S, Jamison JC, Tripodi E (2021). Socio-demographic factors associated with self-protecting behavior during the Covid-19 pandemic. Journal of Population Economics.

[ref-42] Pathania AS, Prathipati P, Abdul BA, Chava S, Katta SS, Gupta SC, Gangula PR, Pandey MK, Durden DL, Byrareddy SN, Challagundla KB (2021). COVID-19 and cancer comorbidity: therapeutic opportunities and challenges. Theranostics.

[ref-43] Pham QD, Stuart RM, Nguyen TV, Luong QC, Tran QD, Pham TQ, Phan LT, Dang TQ, Tran DN, Do HT, Mistry D, Klein DJ, Abeysuriya RG, Oron AP, Kerr CC (2021). Estimating and mitigating the risk of COVID-19 epidemic rebound associated with reopening of international borders in Vietnam: a modelling study. The Lancet. Global Health.

[ref-44] Quilty BJ, Clifford S, Flasche S, Eggo RM, Group2 C nCoV working (2020). Effectiveness of airport screening at detecting travellers infected with novel coronavirus (2019-nCoV). Eurosurveillance.

[ref-45] Regev-Yochay G, Gonen T, Gilboa M, Mandelboim M, Indenbaum V, Amit S, Meltzer L, Asraf K, Cohen C, Fluss R, Biber A, Nemet I, Kliker L, Joseph G, Doolman R, Mendelson E, Freedman LS, Harats D, Kreiss Y, Lustig Y (2022). Efficacy of a fourth dose of covid-19 mRNA vaccine against Omicron. New England Journal of Medicine.

[ref-46] Reis J, Buguet A, Román GC, Spencer PS (2022). The COVID-19 pandemic, an environmental neurology perspective. Revue Neurologique.

[ref-47] Tan JB, Cook MJ, Logan P, Rozanova L, Wilder-Smith A (2021). Singapore’s pandemic preparedness: an overview of the first wave of COVID-19. International Journal of Environmental Research and Public Health.

[ref-48] Taylor L (2021). Covid-19: spike in cases in Chile is blamed on people mixing after first vaccine shot. BMJ.

[ref-49] Times of Israel (2023a). Government extends tourist entry ban for additional week. The Times of Israel. https://www.timesofisrael.com/liveblog_entry/government-extends-tourist-entry-ban-for-additional-week/.

[ref-50] Tran TPT, Le TH, Nguyen TNP, Hoang VM (2020). Rapid response to the COVID-19 pandemic: Vietnam government’s experience and preliminary success. Journal of Global Health.

[ref-51] Viboud C, Bjørnstad ON, Smith DL, Simonsen L, Miller MA, Grenfell BT (2006). Synchrony, waves, and spatial hierarchies in the spread of influenza. Science.

[ref-52] Wang X, Shi L, Zhang Y, Chen H, Sun G (2021a). Coping with COVID-19: core elements of lockdown Wuhan City policy. Journal of Health Care for the Poor and Underserved.

[ref-53] Wang X, Shi L, Zhang Y, Chen H, Sun G (2021b). Policy disparities in fighting COVID-19 among Japan, Italy, Singapore and China. International Journal for Equity in Health.

[ref-54] Wang K, Wong ELY, Ho KF, Cheung AWL, Chan EYY, Wong SYS, Yeoh EK (2021c). Unequal availability of workplace policy for prevention of coronavirus disease 2019 across occupations and its relationship with personal protection behaviours: a cross-sectional survey. International Journal for Equity in Health.

[ref-55] World Health Organization (WHO) (2022). WHO Coronavirus (COVID-19) Dashboard. https://covid19.who.int.

[ref-56] Yang C, Ma QY, Zheng YH, Yang YX (2020). Transmission routes of 2019-novel coronavirus (2019-nCoV). Zhonghua Yu Fang Yi Xue Za Zhi.

[ref-57] Yechezkel M, Weiss A, Rejwan I, Shahmoon E, Ben-Gal S, Yamin D (2021). Human mobility and poverty as key drivers of COVID-19 transmission and control. BMC Public Health.

