# Supplementary material for: The impact of containment policy and mobility on COVID-19 cases through structural equation model in Chile, Singapore, South Korea and Israel
Source: PeerJ. 2023 Aug 1;11:e15769. doi: 10.7717/peerj.15769 (PMC10402700; doi:10.7717/peerj.15769)

**Appendix 1 Measurement model modification**

We need to modify the measurement model corresponding to the latent variables of the whole model to ensure that the fitness of the whole model reaches the standard.

1. Containment policies
2. before modified


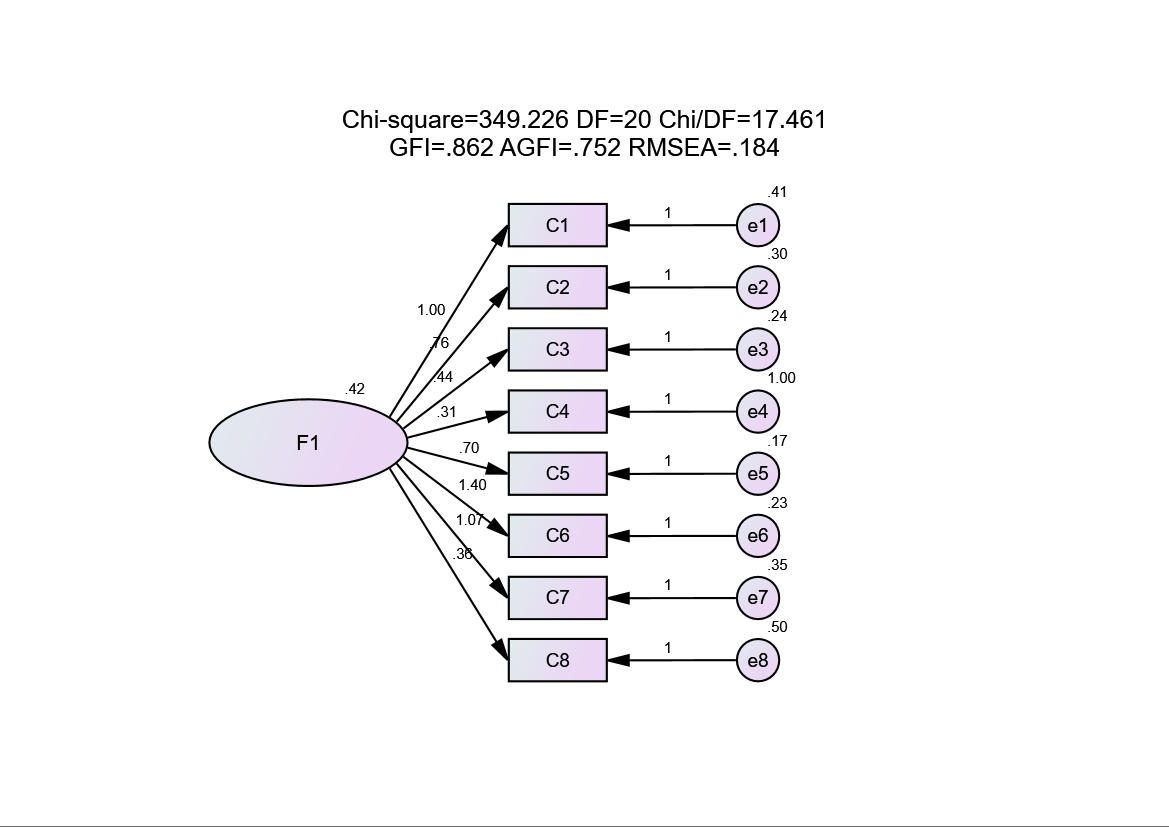


1. after modified


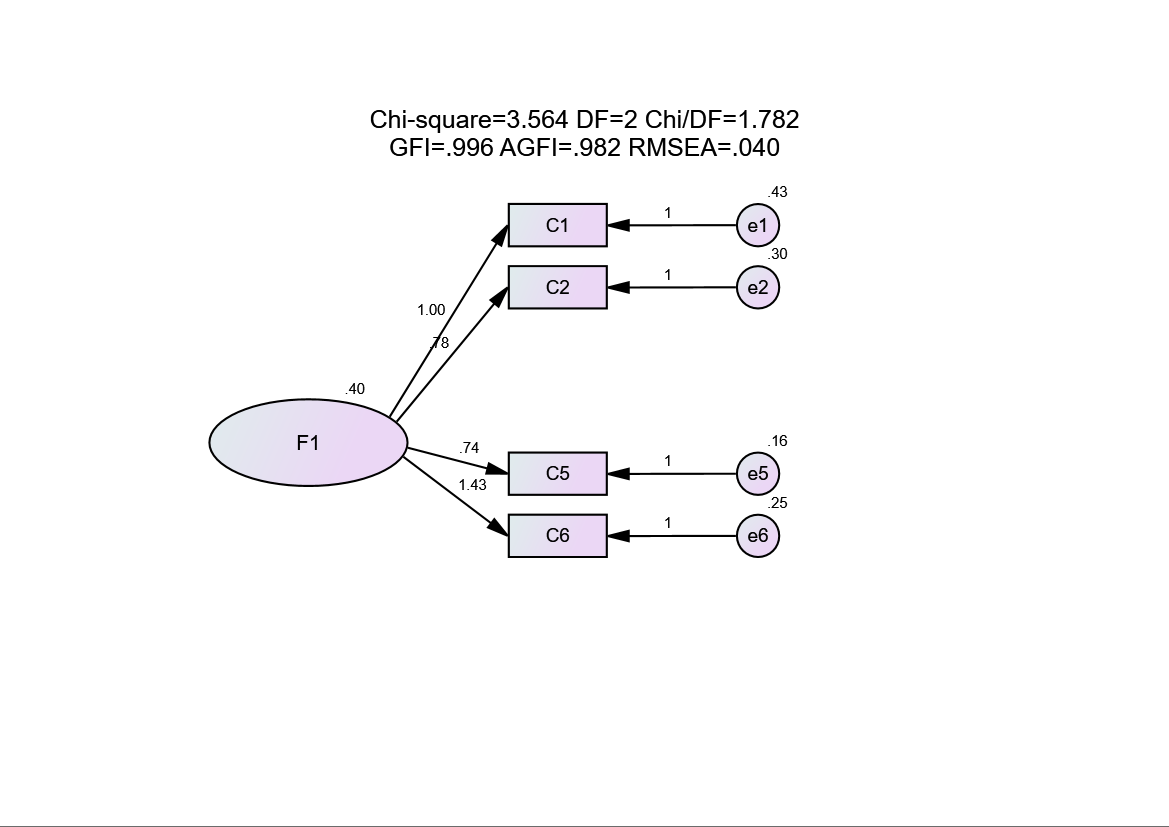


1. mobility
2. before modified


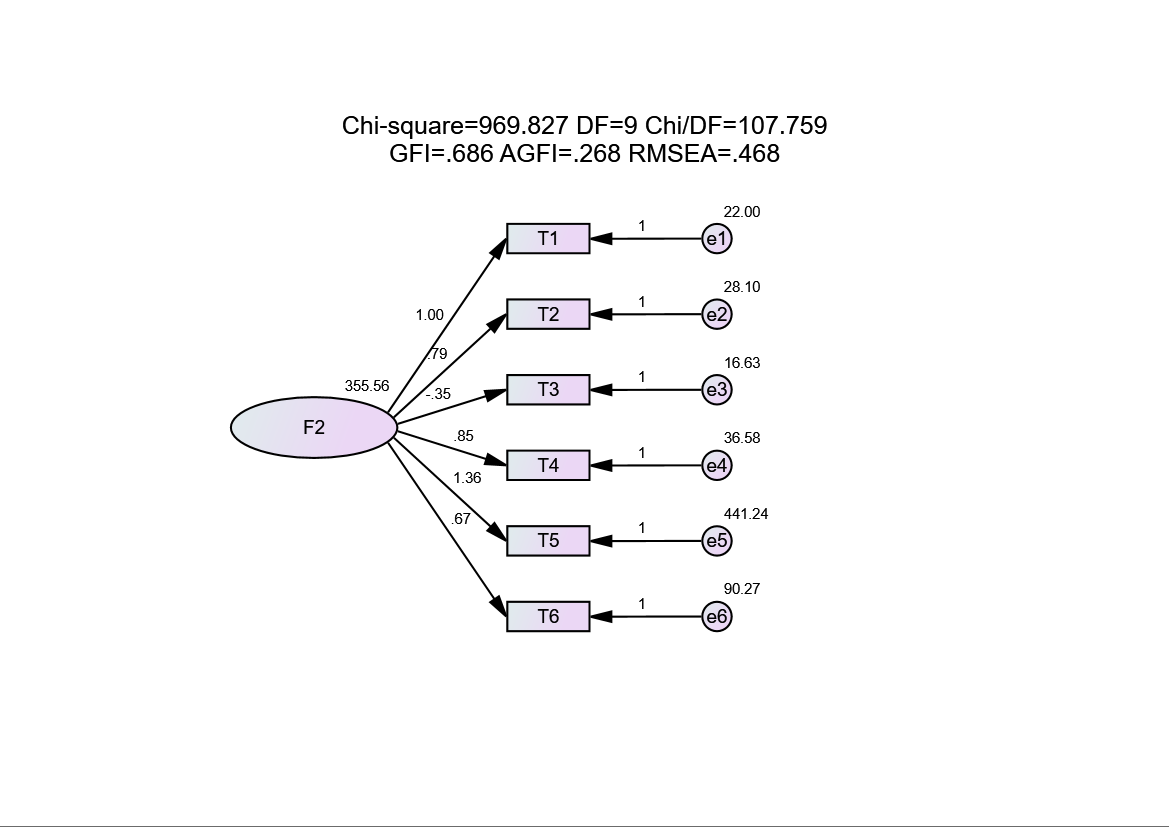


2) after modified


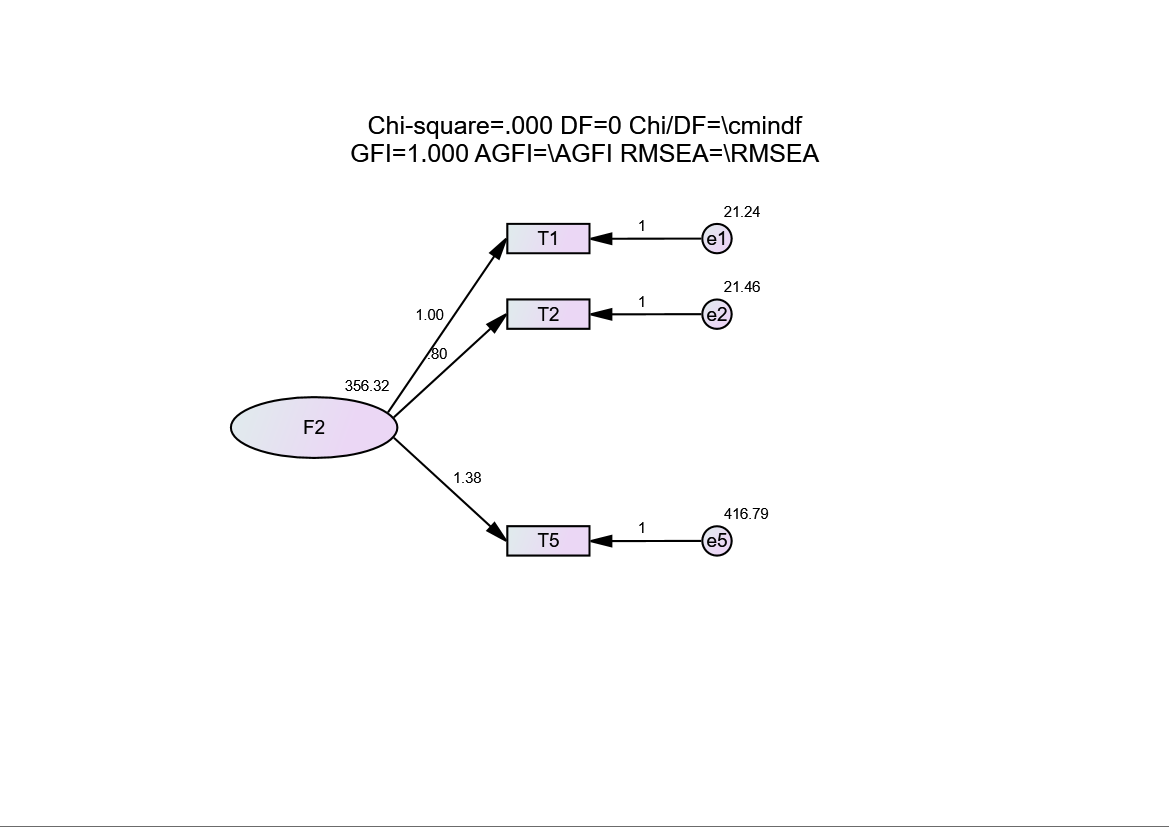


1. COVID-19 cases
2. before modified


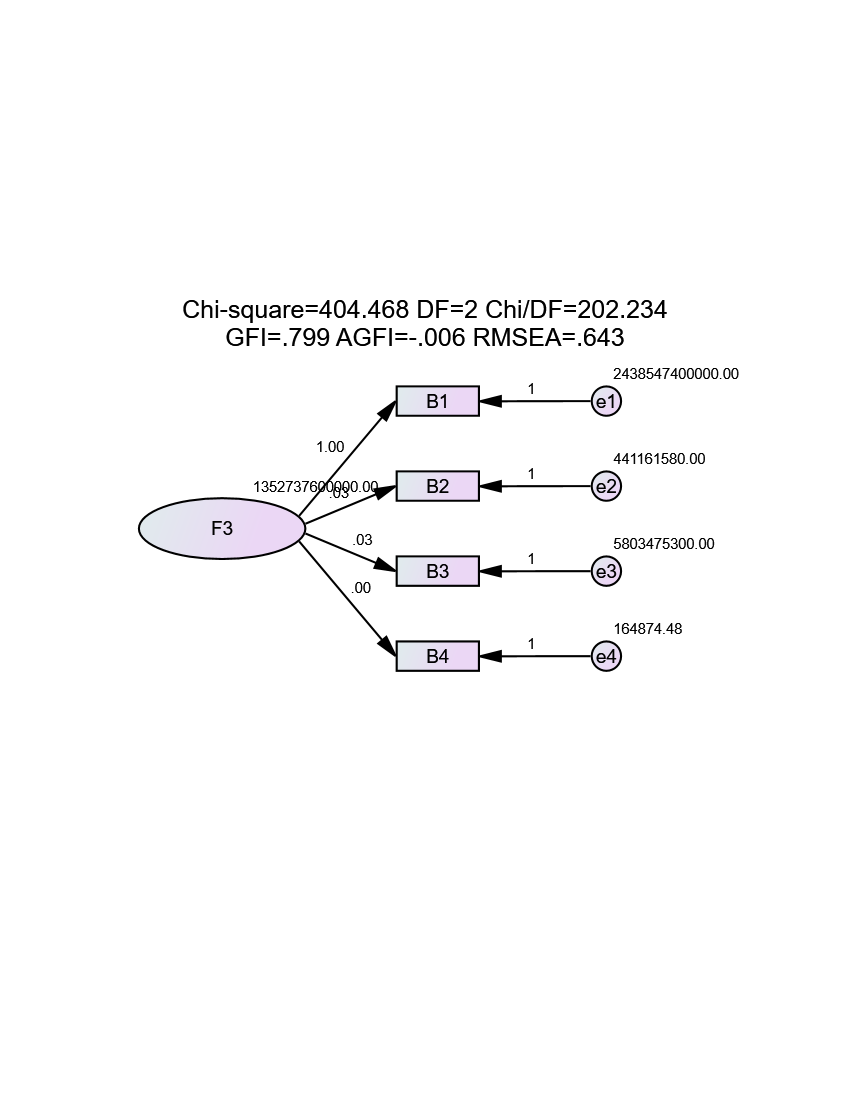


2) after modified


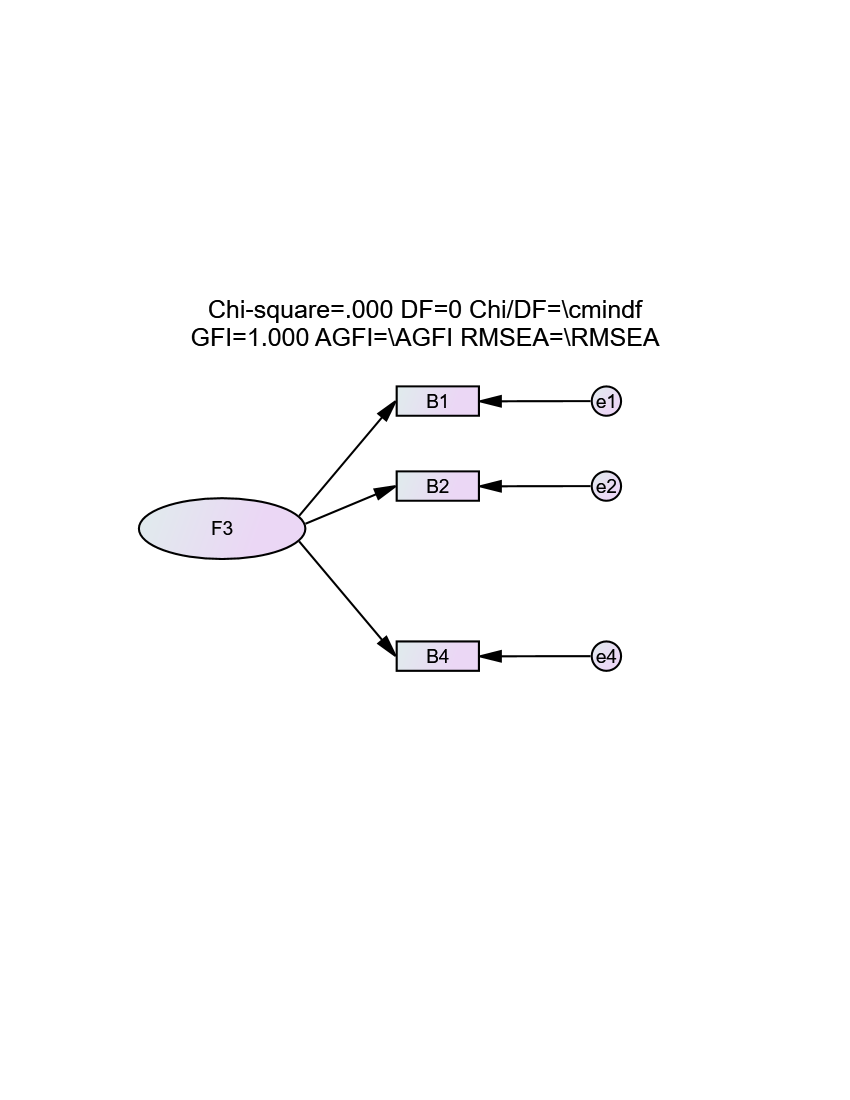

Supplement: Appendix S1 [file peerj-11-15769-s002.docx]
